# Mapping a Type 1 FHB resistance on chromosome 4AS of *Triticum macha* and deployment in combination with two Type 2 resistances

**DOI:** 10.1007/s00122-015-2542-9

**Published:** 2015-06-04

**Authors:** C. Burt, A. Steed, N. Gosman, M. Lemmens, N. Bird, R. Ramirez-Gonzalez, S. Holdgate, P. Nicholson

**Affiliations:** John Innes Centre, Norwich Research Park, Norwich, NR4 7UH UK; IFA-Tulln, University of Natural Resources and Life Sciences, Konrad Lorenz Strasse 20, 3430 Tulln, Austria; The Genome Analysis Centre, Norwich Research Park, Norwich, NR4 7UH UK; RAGT, Grange Road, Ickleton, Essex, CB10 1TA UK

## Abstract

**Key message:**

**Markers closely flanking a Type 1 FHB resistance have been produced and the potential of combining this with Type 2 resistances to improve control of FHB has been demonstrated.**

**Abstract:**

Two categories of resistance to Fusarium head blight (FHB) in wheat are generally recognised: resistance to initial infection (Type 1) and resistance to spread within the head (Type 2). While numerous sources of Type 2 resistance have been reported, relatively fewer Type 1 resistances have been characterised. Previous study identified a Type 1 FHB resistance (*QFhs.jic*-*4AS*) on chromosome 4A in *Triticum macha*. Little is known about the effect of combining Type 1 and Type 2 resistances on overall FHB symptoms or accumulation of the mycotoxin deoxynivalenol (DON). *QFhs.jic*-*4AS* was combined independently with two Type 2 FHB resistances (*Fhb1* and one associated with the 1BL/1RS translocation). While combining Type 1 and Type 2 resistances generally reduced visual symptom development, the effect on DON accumulation was marginal. A lack of polymorphic markers and a limited number of recombinants had originally prevented accurate mapping of the *QFhs.jic*-*4AS* resistance. Using an array of recently produced markers in combination with new populations, the position of *QFhs.jic*-*4AS* has been determined to allow this resistance to be followed in breeding programmes.

**Electronic supplementary material:**

The online version of this article (doi:10.1007/s00122-015-2542-9) contains supplementary material, which is available to authorized users.

## Introduction

Fusarium head blight (FHB) of wheat is caused by several fungal species. *Fusarium graminearum* is the major pathogen worldwide, but *F. culmorum* tends to predominate in maritime regions, and F*. avenaceum* and *F. poae* are also frequently associated with FHB in Northern Europe. Although FHB may cause large reductions in grain yield and baking quality, a greater threat is posed by mycotoxins which contaminate infected grain and pose a risk to human and animal health. *F. graminearum* and *F. culmorum* both produce trichothecene mycotoxins such as deoxynivalenol (DON) and nivalenol (NIV). In addition to the true *Fusarium* species, two *Microdochium* species, *M. majus* and *M. nivale*, also cause FHB and are particularly prevalent where cooler, wetter conditions prevail, such as the UK. In contrast to *F. graminearum* and *F. culmorum*, neither *Microdochium* species are known to produce mycotoxins, but they may still cause significant reductions in grain quality and yield, particularly in cool wet summers when it may outcompete the *Fusarium* species (Nicholson et al. 2003). Both the non-toxin producing *Microdochium* species and the toxin producing *Fusarium* species can initially infect and colonise wheat spikelets, however, only *Fusarium* species will spread through the rachis to infect adjacent spikelets. Additionally, it has been shown that although *F. graminearum* mutants that do not produce DON can colonise wheat heads, they are not able to spread from the point of infection through the rachis (Bai et al. 2002). This suggests that DON is not required for initial infection but is a virulence factor that is necessary for disease spread through the head.

Crop management and agrochemical measures are only partly effective in controlling the disease and therefore the development of FHB resistant varieties is important for disease control and the prevention of mycotoxin contamination. Resistances have been identified in a variety of sources including from Asia (e.g. Sumai-3) (Bai et al. 1999; Waldron et al. 1999), South America (e.g. Frontana) (Schroeder and Christensen 1963; Steiner et al. 2004) and Europe (e.g. Arina) (Snijders 1990). Inheritance of resistance to FHB in wheat is quantitative with a large volume of literature identifying more than 100 quantitative trait loci (QTL) for resistance (Buerstmayr et al. 2009). Several forms of resistance to FHB have been postulated but resistance is generally differentiated into two types: Type 1 (resistance to initial infection) and Type 2 (resistance to spread within the head) (Schroeder and Christensen 1963). The majority of resistance QTL identified confer type 2 resistance (Buerstmayr et al. 2009). This includes the potent 3BS QTL derived from Sumai-3, *Qfhs.ndsu*-*3BS* (Anderson et al. 2001), which was subsequently mapped as a single Mendelian gene termed *Fhb1* (Cuthbert et al. 2006), and a QTL identified on chromosome 1B that is thought to be located on or closely linked to the 1BL-1RS wheat-rye translocation (Ittu et al. 2000; Shen et al. 2003; Schmolke et al. 2005). Type 1 resistance is considered to be advantageous, because it confers resistance to colonisation both by toxin producing *Fusarium* species and non-toxin producing *Microdochium* species. However, it is difficult to identify and select for Type 1 resistance as the presence of this form of resistance must be inferred following assessment by both single spikelet (point) inoculation to assess Type 2 resistance and spray inoculation to assess both Type 1 and Type 2 resistance (Mesterházy et al. 2008). Type 1 resistance QTLs that have been identified include QTL located on chromosome 5A (Buerstmayr et al. 2003; Lin et al. 2006; Steiner et al. 2004), 4B (Lin et al. 2006) and 4A (Steed et al. 2005).

Resistance to FHB has also been described within related species of wheat. In particular, FHB resistance has been identified in *T. macha*, a hulled hexaploid wheat endemic in the Caucasus region (Dardis and Walsh 2003; Gilbert and Tekauz 2000). FHB resistance QTLs were identified in *T. macha* on chromosomes 2A, 2B, 5A and 5B using backcross derived recombinant inbred lines (Buerstmayr et al. 2011) and on chromosome 4A using a set of single chromosome substitution lines (Steed et al. 2005). The *T. macha* 4A resistance was shown to confer Type 1 resistance as it was clearly observed from spray inoculations but was not evident following point inoculation. Importantly, this resistance was shown to reduce both visual disease symptoms and levels of DON, suggesting that it may be useful for deployment in elite varieties to provide protection against FHB. This resistance was mapped as a single gene to 4AS using a double haploid (DH) population, where it co-segregated with the SSR marker Gwm165 and was named *QFhs.jic*-*4AS*. However, the limited number of recombinants (43 lines) combined with a lack of polymorphic distal flanking markers prevented accurate localisation of the QTL (Steed et al. 2005).

More than 100 QTL for FHB resistance have been identified and reported in wheat, as reviewed by Buerstmayr et al. (2009). To provide a high level of resistance to FHB in wheat, marker assisted selection (MAS) of these QTL can be used to pyramid these resistances into an agronomically desirable background. This approach relies on the selection of resistances that function additively to confer an enhanced level of resistance when combined together. It is possible that combining Type 1 resistances such as the *T. macha* 4AS resistance with Type 2 resistances such as the 1B QTL (associated with the 1BL-1RS wheat-rye translocation) and the major 3B QTL (*Fhb1*), may provide an additive effect restricting both initial infection and subsequent spread of the pathogen along the rachis.

In the present study, we tested combinations of the 1B, 3B and 4A FHB resistance QTL, as outlined above, in a susceptible UK wheat background (Hobbit-‘sib’) to examine if pyramiding FHB resistances will confer additional resistance, both in terms of visual disease symptoms and DON content. In addition, we utilised a 288 line F_4_ population developed from the susceptible parent Hobbit ‘sib’ and the resistant line DH81, previously developed by Steed et al. (2005), to refine the localisation of the 4AS *T. macha* Type 1 resistance and to identify SNP markers to aid MAS and pyramiding with other FHB resistance QTL by plant breeders.

## Materials and methods

### Plant material and population development

Seed of the highly FHB susceptible UK variety Hobbit ‘sib’ (HS) was obtained from the John Innes Centre (JIC) wheat collection, and seed of the highly resistant variety WEK0609^®^ (Gosman et al. 2005) was provided by Pioneer Hi-Bred International Inc. Previous SSR haplotyping has suggested that this variety has a number of QTL providing FHB resistance, including *Fhb1* on chromosome 3B and the 1BL-1RS associated resistance (Gosman et al. 2007). Seed was also obtained of the single chromosome substitution line Hobbit ‘sib’/*T. macha* 4A (HS/Tm4A), and a single chromosome recombinant double haploid line (DH81) previously developed by Steed et al. (2005) from the cross between HS/Tm4A × Hobbit ‘sib’ and shown to possess the FHB QTL.

A single chromosome substitution series was generated for HS × WEK0609^®^ in a Hobbit ‘sib’ background. Single chromosome substitution lines (F_6_) of chromosomes 1B and 3B were crossed to DH81 and the resulting F_2_ progeny were screened for the presence/absence of simple sequence repeat (SSR) alleles associated with the 1B, 3B and 4A resistances (see below). This procedure was used to generate a total of 16 independent ‘QTL combination’ lines with a common susceptible background with: the 4A (three lines), 3B (one line) or 1B (two lines) resistance QTL in isolation; a combination of 4A and 1B QTLs (three lines); a combination of 4A and 3B QTLs (four lines); or lacking any FHB QTL (three lines). These ‘QTL combination’ lines were evaluated for FHB resistance in a polytunnel trial and five field trials across 3 years as detailed below.

DH81 was backcrossed to HS and a population of 288 F_4_ plants was generated. Homozygous recombinant F_4_ lines (39 lines) identified within this population were selfed (F_5_) and then bulked for use in phenotypic evaluations of FHB resistance. F_4_ lines that were recombinant but heterozygous for one or more loci were selfed and the resulting F_5_ individuals genotyped to identify additional homozygous recombinants (39 lines). Seed of individual plants was then bulked for use in phenotypic evaluations of FHB resistance in three field trials and one polytunnel trial as detailed below.

### Simple sequence repeat (SSR) marker analysis

SSR primer sets used were from IPK Gatersleben (Gwm), Wheat Microsatellite Consortium (Wmc), Beltsville Agricultural Research Station (Barc) and INRA (Cfa/Cfd/Gpw), and are described on the GrainGenes website (http://wheat.pw.usda.gov/cgi-bin/graingenes/). PCR conditions were as described by (Bryan et al. 1997).

To identify the presence/absence of the 3B, 1B and 4A QTLs in QTL combination lines, the following SSR probes were used: for 3BS, Gwm389, Gwm533 and Gwm493 (Anderson et al. 2001); for 1B, Barc008 and Gwm018 (Shen et al. 2003); and for 4A, Gwm165, Gwm601 and Gwm610 (Steed et al. 2005).

To identify SSR markers for mapping the *T. macha* 4A resistance, HS and DH81 were screened with 39 publically available SSR markers that were reported to be located on chromosome 4A, to identify polymorphic and co-dominant markers. Polymorphic SSR markers (Table S1) were applied to the HS × DH81 F_4_ population and the resulting F_5_ recombinant lines. DNA extractions, PCR conditions and product size determination were conducted as described by Burt et al. (2011).

### Single nucleotide polymorphism (SNP) analysis

The parent lines of the population (HS and DH81) and the single chromosome substitution line HS/Tm4A were screened at LGC Genomics (www.lgcgenomics.com) with a wheat SNP panel using their proprietary KBioscience Competitive Allele-Specific Polymerase chain reaction (KASP) genotyping technique. This SNP panel was developed in conjunction with the University of Bristol and contains over 5000 validated SNP assays (Allen et al. 2013). Polymorphic markers were identified to provide an even coverage of chromosome 4AS and primer sets obtained (Table S1) to apply to the HS × DH81 F_4_ population and the resulting F_5_ recombinant lines.

To identify additional 4AS polymorphisms, the parent lines and HS/Tm4A were run on the iSelect 90 k wheat SNP chip (Wang et al. 2014) at the University of Bristol Genomics Facility (http://www.bristol.ac.uk/biology/research/transcriptomics/). The analysis of alleles was conducted using Genome Studio Data Analysis Software from Illumina (http://www.illumina.com/informatics/sequencing-microarray-data-analysis/genomestudio.ilmn) with a cluster file created by Wang et al. (2014) that was trained on a diversity panel of wheat landraces. The sequence of the polymorphic markers was aligned to the flow-sorted scaffolds from the International Wheat Genome Sequencing Consortium (IWGSC) chromosome survey sequence, [available from EnsemblPlants, release 21 (Kersey et al. 2012)] using BLAT (Kent 2002). A BioRuby script (Goto et al. 2010) was used to select the alignment with the highest score and used to infer the likely chromosomal location. Selected iSelect SNPs on 4AS and other chromosomes containing *T. macha* introgressions were converted into KASP assays by identifying homoeologue SNPs from the survey sequence data and using these in conjunction with the varietal SNPs to design homoeologue specific KASP assays.

QTL combination lines were additionally screened for the presence of the 3B QTL using an *Fhb1* linked KASP assay wMAS000008 to confirm the presence of this resistance as determined by SSR markers. This marker was previously developed by Gina Brown-Guedira (USDA) as part of a panel of KASPs for MAS of agronomically important genes in wheat (http://www.cerealsdb.uk.net/cerealgenomics/CerealsDB/kasp_download.php?URL).

DNA was extracted from all samples as described by Burt et al. (2011), quantified using a NanoDrop 2000 spectrophotometer (Thermo Scientific) and diluted to 10 ng/ul in sterile distilled water for use in KASP–SNP PCR reactions. An 8.112 µl reaction volume consisted of 4 µl of DNA, 4 µl KASP reaction mix (LGC Genomics), and 0.112 µl assay mix (containing 12 µM each allele-specific forward primer and 30 µM reverse primer). The following PCR conditions were used: 15 min at 94 °C; 10 touchdown cycles of 20 s at 94 °C, 60 s at 65–57 °C (decreasing 0.8 °C per cycle); and 26–35 cycles of 20 s at 94 °C, 60 s at 57 °C. Fluorescence detection of the reactions was performed using a Bio-Rad CFX96 real-time PCR machine to conduct end point allelic discrimination with CFX Manager 3.1 software (Bio-Rad Laboratories).

### Conserved orthologous sequence (COS), expressed sequence tag SSR (EST-SSR) and SSR marker analysis

Sequences for the polymorphic wheat genotyping panel SNPs and the polymorphic iSelect SNPs were aligned to the *Brachypodium*, rice and Sorghum genomes using Phytozome v9.1 (www.phytozome.net) to identify the orthologous loci in these species, where present. The Brachypodium sequence corresponding to the region of interest on wheat 4AS was visually examined using the Brachypodium (Bd21) Genome Browser (http://www.modelcrop.org/cgi-bin/gbrowse/brachyv1/) to identify thirty-three COS markers aligned to the region. These markers were previously developed by Dr. Simon Griffiths and Michelle Leverington-Waite at the John Innes Centre.

Expressed sequence tag-derived microsatellite (EST-SSRs) for comparative mapping in wheat, barley and rice were previously identified by La Rota et al. (2005). From these, primers for a set of 26 EST-SSRs were identified in the rice region orthologous to 4AS in wheat. The 33 COS and 26 EST-SSR markers were tested on the parent lines (HS and DH81) to identify polymorphisms on 4AS located in the region of the resistance. Polymorphic markers (Table S1) were applied to the HS × DH81 F_4_ population and the resulting F_5_ recombinant lines.

DNA extractions and PCR reactions were prepared as described by Burt et al. (2011). PCR amplification was conducted using a touchdown programme consisting of a denaturing step of 95 °C for 10 min; 16 touchdown cycles of 95 °C for 15 s, 58 °C for 1 min decreasing 0.5 °C per cycle, 72 °C for 1 min; then 30 cycles of 95 °C for 15 s, 50 °C for 15 s and 72 °C for 1 min. Samples were run on an ABI 3700 capillary sequencer (Applied Biosystems) and the output data were analysed using Peak Scanner v1.0 (Applied Biosystems) to determine the product size of the amplicons. If no polymorphism was detected using this method, then products were examined by single-strand conformation polymorphism (SSCP) assay (Martins-Lopes et al. 2001) using Sequa GelMD (National Diagnostics, UK Ltd.) and visualised by silver staining (Bassam et al. 1991).

### FHB resistance phenotyping of the 1B, 3B and 4A QTL combination lines

16 lines representing the 5 QTL combination categories [1B, 3B, 1B and 4A, 3B and 4A, and Null (none)] were entered into five independent field trials across 2 years. These were: John Innes Centre, Norfolk, UK (JIC) and Tulln, Austria in 2011; and JIC, Tulln, and Church Farm, Bawburgh, Norfolk, UK (CF) in 2012.

These field experiments were conducted in a randomised complete block design with four and two replicate blocks per line in the UK and Austria, respectively. Trials in the UK were inoculated with a highly virulent DON-producing *F. culmorum* isolate (Fu42), whilst in the trials in Austria, a highly aggressive *F. graminearum* isolate (IFA66) was used. In the UK, plants were spray inoculated with a conidial suspension (1 × 10^4^ ml^−1^) amended with 0.05 % Tween20 at mid anthesis [growth stage (GS) 65, (Zadoks et al. 1974)] using a knapsack sprayer (150 ml m^−2^). Plants were mist irrigated for a minimum of 72 h post inoculation to maintain high humidity. The inoculation was repeated after an interval of 3 days. Disease was assessed as % infection within each plot at four time intervals post infection (dpi) to follow disease development in each trial. The area under the disease progress curve (AUDPC) was calculated from percentage infection at each time point to provide an integrated measure of disease.

Additionally, an experiment was conducted in an unheated polytunnel at JIC in 2010 with 4 replicate plants per line arranged in a randomised block design on capillary matting. Inoculum was prepared as described above and plants were inoculated at GS65 with a conidial suspension (1 × 10^4^ ml^−1^) amended with 0.05 % Tween20 at GS65 using a held–held sprayer. Following spray inoculation, plants were visually assessed for disease on the basis of percentage of spikelets infected per head at 10, 14 and 18 dpi (%FHB). Percentage spikelet infection at 18 dpi was used as a measure of disease severity. The area under the disease progress curve (AUDPC) was again calculated to provide an integrated measure of disease development.

In Austria, all the plots (2 rows, 80 cm long) were repeatedly sprayed with 100 ml m^−2^ of a 1 × 10^4^ conidia/ml macroconidial suspension using a backpack sprayer. The first inoculation was done 2 days before the earliest line flowered and the treatment was repeated (in total 6 applications with 2 day time intervals) until the last line reached full anthesis. During and after each inoculation cycle, the crop canopy was kept wet with a computer controlled mist irrigation system for 20 h. GS 65 was assessed for each line individually. Ten days after mid anthesis, visual disease assessment started and was repeated on day 14, 18, 22 and 26 after mid anthesis. The percentage of visually diseased spikelets was recorded with which the AUDPC was calculated.

Grain samples were taken from the JIC and Tulln field trials in both 2011 and 2012, and the polytunnel trial in 2010. Samples were milled and DON was extracted in 10 % methanol and DON content was assessed using the Ridascreen Fast DON™ (R-Biopharm Rhone Ltd.) enzyme linked immuno-assay (ELISA) according to the manufacturer’s instructions as described previously (Gosman et al. 2005).

### FHB resistance phenotyping of the HS × DH81 population

In total, 78 recombinant lines were selected from the HS × DH81 F_4_ population for use in the current study. Thirty nine stable recombinant F_5_ lines initially identified and generated from the F_4_ population were assessed for FHB resistance in a field trial at JIC in 2012. The FHB resistance of all 78 recombinant F_5_ lines was assessed during the summer of 2013 in two independent field trials; one at CF and one at JIC. These field experiments were conducted in a randomised complete block design with three replicate plots per line. All trials were inoculated with a highly virulent DON-producing *F. culmorum* isolate (Fu42) and conducted as described above. Disease was assessed as % infection within each plot at 16, 22, 25 and 30 days post infection (dpi). The area under the disease progress curve (AUDPC) was again calculated to provide an integrated measure of disease and % infection at 30 dpi was used as a measure of disease severity (%FHB).

The 39 stable recombinant HS × DH81 F_5_ lines initially identified and generated from the F_4_ population were assessed for FHB resistance in 2013 at JIC in an unheated polytunnel with capillary matting irrigation. Fifteen plants per line were arranged in a randomised complete block design with 4 blocks (3–4 plants per line within each block). Inoculations were conducted and plants were scored as described for the above polytunnel trial.

### Statistical analysis

Analysis of variance (ANOVA) using a general linear model (GLM) was performed on AUDPC scores and DON contents (ppm) from the independent FHB resistance experiments for the 1B, 3B and 4A QTL combination lines to assess variation due to QTL class and experiment × QTL class interaction. Means were predicted across the relevant lines for each QTL class, and were subsequently compared using Fisher’s least significant difference test.

ANOVA using a GLM was also performed on %FHB and AUDPC scores from the four phenotyping experiments for the HS × DH81 population to assess variation due to line and experiment × line interaction. This was conducted separately for 39 line experiments (using all data: 2012 JIC, 2013 JIC, 2013 CF and 2013 Polytunnel) and for the 78 line experiments (2013 JIC and 2013 CF) to provide balanced datasets for analysis. Predicted mean disease scores were calculated for the lines within the GLMs. In addition, GLMs were fitted for each experiment individually and predicted mean scores for each line calculated within the models to account for variation due to field block. Means calculated across experiments and means calculated within experiments were used in subsequent QTL analyses.

All GLMs were conducted in Genstat v. 15.2. Broad sense heritability across experiments was estimated from the ANOVA using the formula: $H = \, {{\sigma_{\text{G}}^{{^{ 2} }} } \mathord{\left/ {\vphantom {{\sigma_{\text{G}}^{{^{ 2} }} } {\left[ {\sigma_{\text{G}}^{{^{ 2} }} + \, \left( {\sigma_{\text{GE}}^{{^{ 2} }} /{\text{E}}} \right) \, + \, \left( {\sigma_{\text{e}}^{{^{ 2} }} /{\text{rE}}} \right)} \right]}}} \right. \kern-0pt} {\left[ {\sigma_{\text{G}}^{{^{ 2} }} + \, \left( {\sigma_{\text{GE}}^{{^{ 2} }} /{\text{E}}} \right) \, + \, \left( {\sigma_{\text{e}}^{{^{ 2} }} /{\text{rE}}} \right)} \right]}}$, with $\sigma_{\text{G}}^{{^{ 2} }}$ the genetic variance, $\sigma_{\text{GE}}^{{^{ 2} }}$ the genotype × experiment interaction variance, $\sigma_{\text{e}}^{{^{ 2} }}$ the residual variance, E the number of experiments, and r the number of replicates per genotype (Nyquist 1991).

### Map construction and QTL analysis

A genetic linkage map of chromosome 4A was constructed using 14 LGC KASP wheat SNPs, 2 iSelect SNP derived KASPs, 3 SSRs and 2 EST-SSRs applied to the DNA from 288 F_4_ lines. The linkage analysis was performed in Joinmap (version 3.0) (Van Ooijen and Voorips 2001), using 0.4 as the maximum recombination fraction and 5.0 as the logarithm of the odds ratio (LOD), and the linkage map was drawn using MapChart (Voorrips 2002).

Predicted mean AUDPC and %FHB scores were calculated for the 39 HS × DH81 F_5_ recombinant lines across all 4 experiments (2012 JIC, 2013 JIC, 2013 CF and 2013 Polytunnel), and for all of the 78 lines HS × DH81 F_5_ recombinant lines across the two experiments in which all lines were included (2013 JIC and 2013 CF). These predicted means were then used alongside marker data from the same lines in simple interval mapping (SIM) in Genstat v.15.2. Significance of QTL were presented as *p* values on a log10 scale [−log10(*p*)]. To declare QTL, a −log10(*p*) threshold of 2.342 was determined using the method of Li and Ji (2005) with a genome wide significance level of 0.05.

To check for stability of QTL effects across different trials, predicted mean %FHB and AUDPC scores from the field trials and polytunnel experiment of F_5_ recombinant lines were used alongside marker data from the same lines in a single marker regression analysis to identify QTL locations for each trait within each experiment. A single marker regression analysis was utilised as there were relatively few markers (21) densely spaced on a single linkage group. Markers were only determined to be associated with the phenotype, where *p* < 0.01, to reduce the likelihood of false positives.

## Results

### FHB resistance in the 1B, 3B and 4A QTL combination lines

SSR haplotype data identified 16 lines with the following combinations of QTL: (i) the 3B QTL alone (1 line), (ii) the 1B QTL alone (2 lines), (iii) the 4A QTL alone (3 lines), (iv) 4A and 3B QTLs (4 lines), (v) 4A and 1B QTLs (3 lines), and (vi) ‘Null’ lines (3 lines) as susceptible controls that had been through the crossing procedure but lacked any of the QTL according to SSR haplotype data. The presence or absence of the 3B QTL in the QTL combination lines as predicted by the SSR data was additionally confirmed by wMAS000008.

Data were combined across the lines for each QTL class and also across the trials to conduct an analysis of variance and to predict means for AUDPC and DON contents. Effects of the QTL combination classes were significant for both AUDPC and DON contents (Table 1). However, there was a significant class by trial interaction for both AUDPC and DON contents (*p* < 0.001)) suggesting that there were differences in the relative performance of the lines across the trials. Broad sense heritability estimates were relatively high for AUDPC (0.80) suggesting a high level of stability of the effect of the QTL combinations in different environments. However, DON contents were less stable across environments for the QTL combinations with a heritability of 0.60 (Table 1).

**Table 1:** Variance components of AUDPC and DON contents (ppm) using general linear modelling over all six experiments for the QTL combination classes

Source of variation	AUDPC		DON (ppm)	
	MS	*F* value	MS	*F* value
QTL class	1,375,279	22.05***	810.22	12.82***
QTL class × experiment	213,389	3.42***	323.35	5.12***
Residual	62,376		63.21	
*H*	0.8		0.6	

*MS* mean squares, *H* broad sense heritability

*** *p* < 0.001

**Fig. 1:**
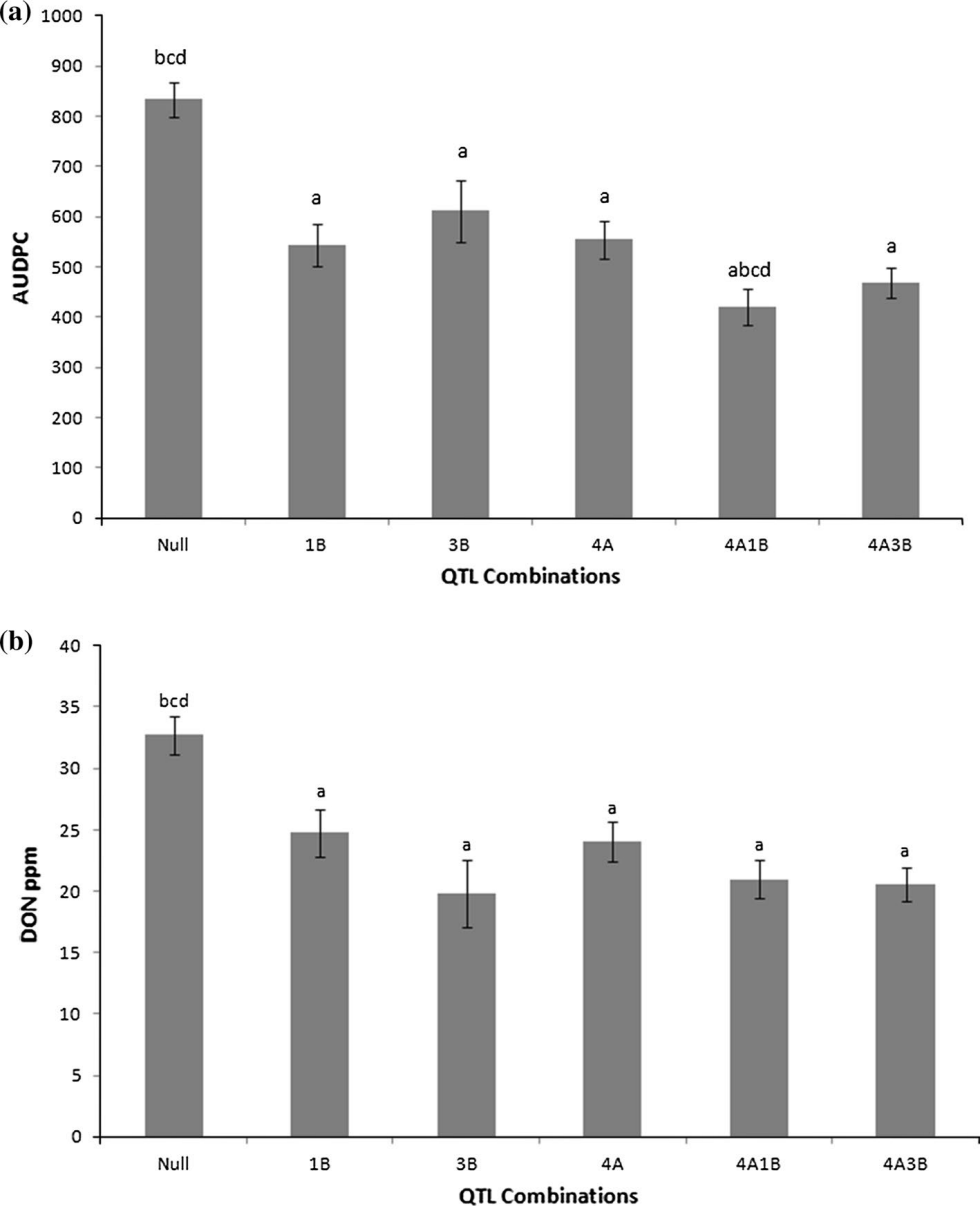
Predicted mean **a** AUDPC scores** b** DON contents of grain samples in ppm for the QTL classes calculated across trials.* Error bars* are all ± standard error of the mean. Predicted are compared using Fisher’s least significant difference test and comparisons demonstrating significant differences (*p* < 0.05) are shown as: *a* from Null; *b* from 1B; *c* from 3B; *d* from 4A

Predicted means for AUDPC and DON contents demonstrated that the 1B, 3B and 4A QTL individually confer a significant reduction (*p* < 0.05) in both visual disease scores and DON levels compared to the ‘Null’ lines without any known resistance QTL (Fig. 1). There was some evidence that combinations of QTLs may have an additive effect on visual disease scores. The combination of 4A and 1B FHB QTLs conferred a significant reduction in visual disease scores compared to either 4A alone or 1B alone (*p* < 0.05) (Fig. 1a). Although the 3B and 4A combined resistance provided some evidence of enhanced disease control compared to the 4A QTL alone, the reduction was not significant (Fig. 1a).

There was no evidence that combining QTL enhances resistance to DON accumulation. Combining the 1B and 4AQTL reduced the amount of DON compared to either QTL in isolation (Fig. 1b), however, these reductions were not significant (*p* > 0.05). The combination of 4A and 3B QTLs provided a similar level of reduction in DON content compared to the 3B QTL alone.

Differences in FHB resistance are often associated with plant height (Srinivasachary et al. 2008). All lines in the current work, however, were of similar height so removing this aspect for consideration.

### HS × DH81 marker analysis, genotyping and map construction

Of the 115 LGC wheat KASP–SNP markers previously shown to be on chromosome 4AS (Allen et al. 2011, 2013), 20 were polymorphic (Table S2). From these, a sub-set of 14 co-dominant and polymorphic SNPs was identified to provide an even coverage of chromosome 4AS on the basis of published SNP maps of the Avalon × Cadenza and Savannah × Rialto populations (Allen et al. 2011, 2013). These 14 SNP markers were applied to the HS × DH81 F_4_ population and also to the resulting F_5_ recombinant lines to confirm the genotypes. The 33 COS and 26 EST-SSR markers were tested on HS and DH81 to identify polymorphisms. Only two EST-SSR markers were polymorphic and these were applied to the HS × DH81 F_3_ population and F_4_ recombinant lines. No COS markers were polymorphic and were therefore not applied to the population (Table S2). Of the 39 SSR markers tested, 9 were polymorphic of which 3 were co-dominant (Wmc48, Gwm192 and Gwm165). One of these, Gwm165, was previously identified by Steed et al. (2005). These three SSR markers were applied to the HS × DH81 F_4_ population and subsequent F_5_ recombinant lines.

**Fig. 2:**
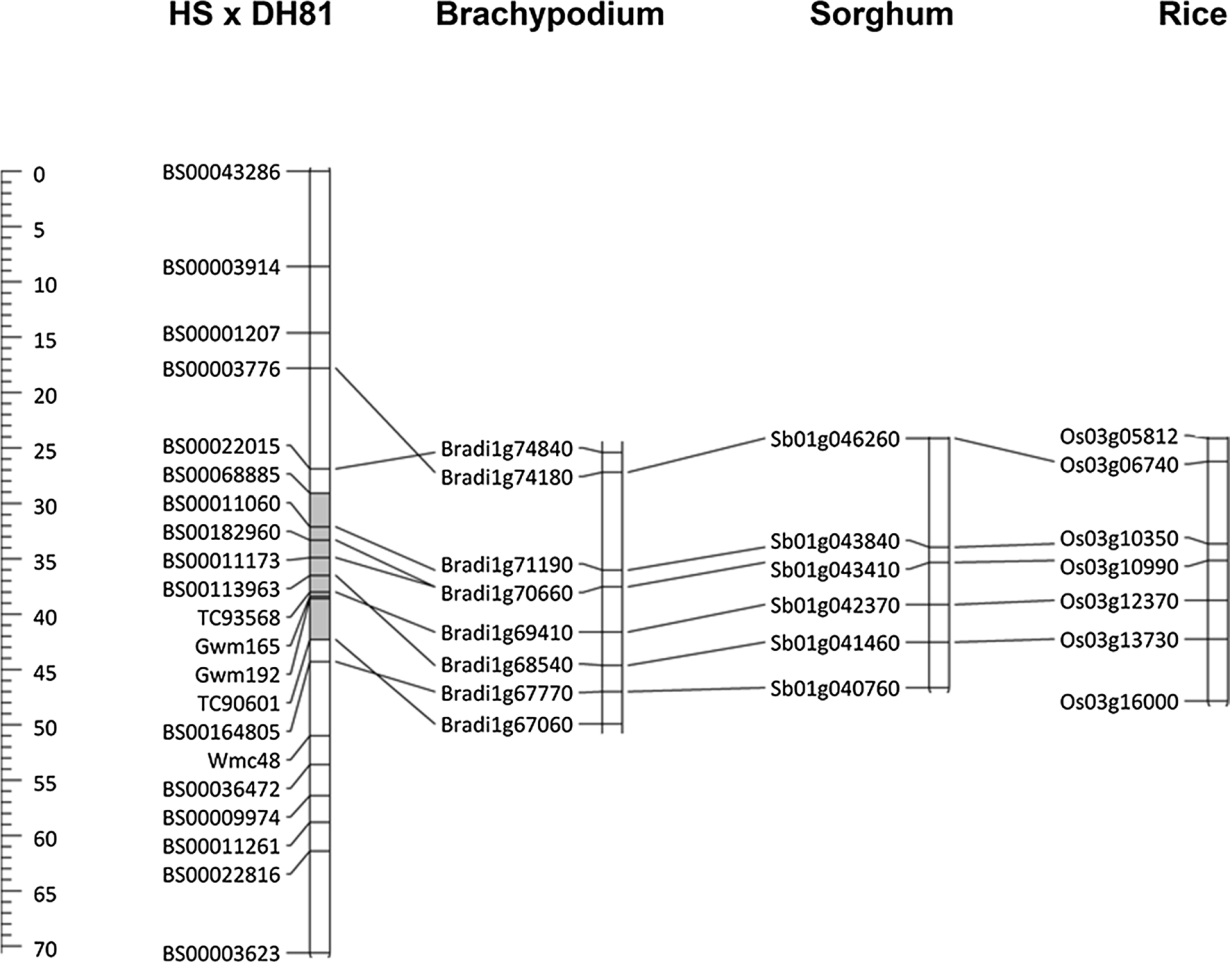
Linkage map of chromosome 4AS of Hobbit ‘sib’ (HS) × DH81 compared to the physical marker locations on Brachypodium chromosome 1, Sorghum chromosome 1 and rice chromosome 3. Genetic map distances in HS × DH81 are indicated in cM on the scale on the *left*. The shaded area indicates the approximate region of the 4A QTL

Eighty-three polymorphic iSelect markers were identified on 4AS. From these, 6 markers were identified by BlastN to have homology to Brachypodium, rice and Sorghum genes within the region orthologous to the QTL location as initially located by the LGC KASP, SSR and EST-SSR markers (Fig. 2). Two markers on 4AS were successfully converted to co-dominant KASP assays (BS00182960 and BS00164805) and were applied to the HS × DH81 F_4_ and F_5_ lines.

In total, 14 LGC wheat KASP SNPs, 2 iSelect derived KASPs, 3 SSRs and 2 EST-SSR markers (Fig. 2) were applied to the 288 lines in the HS × DH81 F_4_ population to construct a genetic map totalling 70.6 cM (Fig. 2). These 21 markers were also applied to each of the 78 recombinant F_5_ lines to confirm the genotypes.

Regions in *Brachypodium*, rice and sorghum were identified with synteny to the *QFhs.jic*-*4AS* region in wheat from BS00022015 to BS00164805 (Fig. 2). Although within this region, there was a high level of gene order conservation between the three reference genomes; this was not completely conserved in the genetic map order from HS × DH81, with the marker pairs BS00164805 and TC90601, TC93568 and BS00113963, and BS00022015and BS00003776 inverted compared to their orthologues. It was not possible to establish co-linearity outside of this region, with only two wheat markers identifying orthologues (BS00022816 orthologous to Bradi1g11550, BS00003914 orthologous to Bradi4g20120).

### HS × DH81 trait analysis

Line effects were significant for both AUDPC and % FHB traits in both 39 and 78 line experiment sets (Table 2). However, there was a significant line by experiment interaction for AUDPC in the 78 line experiments (*p* < 0.001) suggesting that there were differences in the relative performance of the lines across the two trials. Broad sense heritability for both AUDPC and %FHB traits were higher in the 39 line experiments (Table 2). This may reflect a better estimate of the genetic effects when using 4 environments, and additionally, may be influenced by a greater level of environmental control and more detailed scoring of disease in the polytunnel experiment of 39 lines.

**Table 2:** Variance components of FHB resistance traits using general linear modelling for (a) the 78 line experiments (2013 JIC and 2013 CF) and for (b) the 39 line experiments (2012 JIC and 2013 Polytunnel) for lines from the the Hobbit ‘sib’ × DH81 population

Source of variation	AUDPC		% FHB	
	MS	*F* value	MS	*F* value
(a)				
Line	16,430	4.44***	150.71	3.75***
Line × experiment	7336	1.98***	47.78	1.19
Residual	3698		40.14	
*H*	0.55		0.68	
(b)				
Line	32,774	2.87***	1660.1	2.84***
Line × experiment	8588	0.75	299.5	0.51
Residual	11,408		584.6	
*H*	0.74		0.82	

*MS* mean squares, *H* broad sense heritability

*** *p* < 0.001

Predicted means of AUDPC and %FHB for the HS × DH81 recombinant F_5_ lines were plotted in frequency histograms for all experiments (Figure S1). It was not possible to detect a bimodal distribution as identified for the *T. macha* 4A resistance in Steed et al. (2005), with all experiments providing an approximate normal distribution of means.

### HS × DH81 QTL analysis

**Fig. 3:**
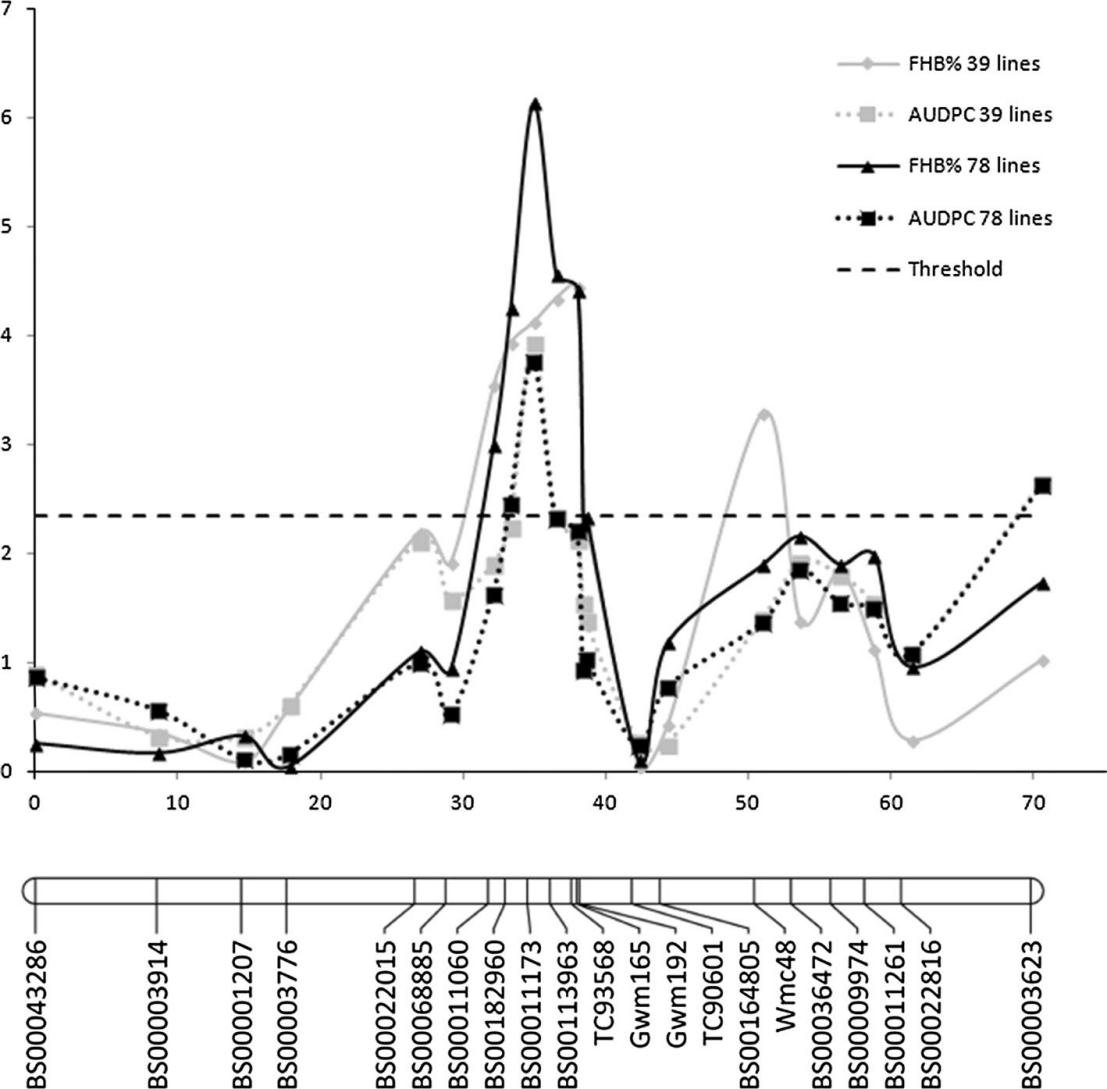
QTL scans for FHB resistance traits, %FHB and AUDPC, using simple interval mapping in the 39 line set and 78 line set of the Hobbit ‘sib’ × DH81 population, aligned to the genetic map for chromosome 4A derived from the population

Significant QTL originating from DH81 and conferring FHB resistance was detected in both the 39 line set and 78 line sets. The peak QTL position was located on the marker BS00011173 for AUDPC in both sets of lines and the same marker was identified as the peak QTL position for %FHB in the 78 line set. A slightly different location at TC93568 was identified as the QTL peak for %FHB in the 39 line set. However, this marker is only 3.1 cM away (Table 3), suggesting that this represents the same genetic effect. The QTL scan shows clear QTL peaks, particularly for %FHB across all 78 lines. It also shows consistent QTL location with LOD scores above the significant threshold for all 4 QTL analyses in the region approximately between 30 and 40 cM (Fig. 3). In addition to the main QTL identified at BS00011173 and TC93568, additional resistance QTL originating from HS were detected in the 39 line set at marker Wmc48 for %FHB and at marker BS00003623 for AUDPC.

**Table 3:** Summary of QTL detected above threshold (−log10(*p*) = 2.324) for FHB resistance traits, %FHB and AUDPC, using simple interval mapping in the 39 line set and 78 line set of the Hobbit ‘sib’ × DH81 population

Sites	Year	Trait	Locus	Position	−log10(*p*)	% Variance explained	Origin
All	2012	%FHB 39 lines	TC93568	38	4.44	30.2	DH81
	2013						
All	2012	%FHB 39 lines	Wmc48	51.0	3.29	22.0	HS
	2013						
All	2012	AUDPC 39 lines	BS00011173	34.9	3.93	26.2	DH81
	2013						
All	2012	AUDPC 39 lines	BS00003623	70.6	2.64	17.6	HS
	2013						
JIC and CF	2013	%FHB 78 lines	BS00011173	34.9	6.13	25.8	DH81
JIC and CF	2013	AUDPC 78 lines	BS00011173	34.9	3.76	11.1	DH81
JIC and CF	2013	AUDPC 78 lines	BS00003623	70.6	2.62	5.7	HS

A single marker regression of individual trial locations identified that the major resistance QTL from DH81 identified in the SIM analysis was consistent across the experiments (Table 4). For AUDPC the single marker regression identified a significant association (*p* < 0.01) between resistance and markers within 3.5 cM of BS00011173 in all trials. Significant associations with %FHB were detected in a region overlapping BS00011173 and TC93568 in three experiments (2013 JIC, 2013 CF and Polytunnel 2013). However, the experiment conducted in 2012 at JIC was less consistent, identifying a QTL at Gwm165, which is only 0.4 cM from TC93568, but also identifying a QTL at BS0006885 and BS00022015, which are 5.8 and 7 cM, respectively, from BS0001173 (Table 4).

**Table 4:** Single marker regression of predicted mean (a) AUDPC scores and (b)  %FHB scores, calculated in general linear models, against chromosome 4A marker scores for recombinant lines from Hobbit ‘sib’ (HS) × DH81 in four independent trials

Marker	cM	% Variance accounted for				Origin
		2012 JIC AUDPC	2013 JIC AUDPC	2013 CF AUDPC	2013 Polytunnel AUDPC	
(a)						
BS00043286	0.0	2.5	1.5	0.2	1	
BS00003914	8.6	0.0	0.4	0.4	0	
BS00001207	14.6	1.0	0	0	0	
BS00003776	17.8	0.0	0.4	0.4	0	
BS00022015	26.9	16.1	3.2	1	3.1	
BS00068885	29.1	15.1	0	0	4.4	
BS00011060	32.1	6.5	3.8	2.4	8.6	
BS00182960	33.3	0.0	4.8	13.6**	14.2	DH81
BS00011173	34.9	8.4	9**	12.0***	11.6	DH81
BS00113963	36.5	4.0	0.6	13.8***	19.4**	DH81
TC93568	38.0	4.2	1.2	9.2**	34.6***	DH81
Gwm165	38.4	19.5**	0	2.2	16.1**	DH81
Gwm192	38.6	11.5	0.3	4.9	18.1**	DH81
TC90601	42.3	0.0	0	0	5.1	
BS00164805	44.3	0.0	0	8.8	10.1	
Wmc48	51.0	0.0	7.9**	0	1.3	HS
BS00036472	53.6	11.2	8.9**	1.1	1.3	HS
BS00009974	56.4	10.0	6.8	0.5	0	
BS00011261	58.8	10.6	4.3	1.8	0	
BS00022816	61.4	9.8	5.8	0	4.8	
(BS00003623)	70.6	13.8	8.3	3.7	0	

Marker	cM	% Variance Accounted For				Origin
		2012 JIC %FHB	2013 JIC %FHB	2013 CF %FHB	2013 Polytunnel %FHB	
(b)						
BS00043286	0.0	5.4	0	0	1	
BS00003914	8.6	0	0	1.1	0	
BS00001207	14.6	3.6	4.4	0	0	
BS00003776	17.8	3.1	0	1.1	2.7	
BS00022015	26.9	15.6**	0	0	0.3	DH81
BS00068885	29.1	18**	3.2	0	1.2	DH81
BS00011060	32.1	4.3	12.1**	6	15.7	DH81
BS00182960	33.3	3	18.4***	19.9***	24**	DH81
BS00011173	34.9	4.8	22.4***	15.5***	18.3***	DH81
BS00113963	36.5	6.1	10.7**	22***	16**	DH81
TC93568	38.0	3.8	10.9**	16.7***	34.6***	DH81
Gwm165	38.4	22.1**	4	7.4**	18.1**	DH81
Gwm192	38.6	15.5**	6.7	12.6**	26.8***	DH81
TC90601	42.3	0	0	0	7.2	
BS00164805	44.3	0	1.3	12.8**	7.9	DH81
Wmc48	51.0	11.9	5	1.5	6	
BS00036472	53.6	10.7	5.1	3.9	0	
BS00009974	56.4	10.3	4	3.9	0	
BS00011261	58.8	8.5	2.8	6.3	0	
BS00022816	61.4	8.3	1.1	0	0	
BS00003623	70.6	9.6	4.3	1.9	0	

** *p* < 0.001; *** *p* < 0.001

Although a QTL from HS conferring a reduction in %FHB was detected at Wmc48, and a QTL from HS conferring a reduction in AUDPC was detected at BS0003623 in the SIM analysis of the 39 line set, the single marker regression of data from individual trials only identified significant associations between this region and disease traits at markers Wmc48 and BS00036472 in the AUDPC trait from the 2013 JIC trial (Table 4). This suggests that this genetic effect is not consistent across environments.

The polytunnel trial found the EST-SSR marker TC93568 accounts for the highest proportion of variation for both AUDPC and %FHB traits, using single marker regression. The greater amount of variation accounted for by markers within the polytunnel trial, compared to the field trials, may be due to a more homogenous environment and/or the more detailed scoring of individual spikelets in this procedure. Steed et al. (2005) conducted all phenotyping in a polytunnel, and this may have assisted the detection of the resistance as a single gene with Mendelian inheritance.

### Background effects in the HS × DH81 population

The wheat KASP panel and the wheat iSelect chip detected the presence of polymorphisms between HS and DH81 on chromosomes 4B and 7A (Table S3). These are likely to be due to remaining *T. macha* introgressions in DH81 that have not been removed by backcrossing. To test if these regions were influencing the phenotype, primers were obtained for the polymorphic wheat SNP panel KASP assays on 4B and 7A. On 4B, the wheat SNP BS00022576 provided a clear assay and was applied to the HS × DH81 F_5_ recombinants. None of the 7A wheat KASP assays provided clear polymorphisms when tested on the population and therefore the iSelect SNP BS00160015 on 7A was converted into a KASP assay that was also applied to the HS × DH81 F_5_ recombinants. Single marker regressions to compare these two markers to AUDPC and  %FHB in the three trials did not identify any significant relationships (*R*^2^ = 0–7.4 %, *p* > 0.05), suggesting that these regions are not influencing the observed phenotypes (Table S3). As for the QTL lines, no difference in plant height were observed within the HS × DH81 population.

## Discussion

Previous genetic studies have suggested that relatively small effect FHB resistances may function additively to confer a higher level of resistance (Anderson et al. 2001; Buerstmayr et al. 2003; Snijders 1990). We combined the Type 2 1B and 3B resistances with the type 1 resistance from *T. macha* 4A (*QFhs.jic*-*4AS*) in a winter wheat background to test for additive effects when combining these resistances. The recurrent parent used in this experiment was Hobbit ‘sib’, a UK winter wheat with a high level of susceptibility. In particular, it contains *Rht*-*D1b*, which has been shown to be highly associated with susceptibility to FHB in numerous studies (Kollers et al. 2013; Srinivasachary et al. 2008, 2009). Despite the high level of susceptibility in the recurrent parent and the high level of disease pressure applied in inoculated, irrigated trials, the 3 resistances all conferred a high level of resistance when deployed individually, reducing both visual disease symptoms and DON content. Both 4A-3B and 4A-1B combinations demonstrated enhanced resistance in terms of reduced visual disease symptoms compared to the individual QTL suggesting that these resistances may function additively to reduce disease. This may be due to the combination of the Type 1 resistance *QFhs.jic*-*4AS*, with the Type 2 resistances *Fhb1* (3B) and the 1B QTL. However, there was no evidence that these combined resistances functioned additively to reduce the amount of DON that was present in wheat grain at harvest. It is possible that more than one type 2 resistance, which act to prevent DON mediated disease spread, are required to provide an additive reduction in DON levels.

The development of varieties with pyramided FHB resistances will be facilitated by increased mapping accuracy and more markers for selection of resistances. *Fhb1* on chromosome 3B and the 1B QTL have been extensively mapped and a number of molecular markers are available for their selection through MAS. However, prior to this study, the map location of *QFhs.jic*-*4AS* was imprecise. Previous efforts to map this resistance have been restricted by a lack of polymorphic markers. Steed et al. (2005) utilised existing SSR and developed novel sequence-specific amplified polymorphism (SSAP) markers, but were not able to identify any distal markers to flank the resistance to facilitate marker assisted selection of the resistance by plant breeders. Developments in SNP technology and the availability of wheat SNPs both through the KASP assays (Allen et al. 2013) and the wheat 90 K iSelect genotyping assay (Wang et al. 2014) enabled saturation of the region surrounding the 4AS QTL. It was therefore possible to identify breeder-friendly KASP markers underlying the QTL region such as BS00011173 and BS00113963. It was also possible to identify distal flanking KASP assay markers such as BS0006885 and BS00022015, and proximal flanking markers such as the iSelect derived KASP BS00164805 and the KASP assay BS00036472 that would be suitable for selection of the region containing *QFhs.jic*-*4AS*. Although the effect of *QFhs.jic*-*4AS* was not potent in trials involving the recombinant lines, it was clear in the QTL combination lines, both alone and in combination with the Type 2 resistances from 1B and 3B.

Previously, Steed et al. (2005) located the *QFhs.jic*-*4AS* as a single gene using visual disease symptoms observed in a polytunnel using a population 43 DH lines. The genetic effect of the region as a whole appears to be relatively large, providing heritability estimates of 0.55–0.82 for the FHB resistance traits recorded (Table 3), and the effect of the QTL was highly significant when studied in the QTL combination lines (Fig. 1) In contrast, in the present study using 78 F_5_ lines from a recombinant population, we were unable to resolve the resistance as a single gene in any experiment or across experiments. However, we were able to locate the resistance quantitatively using SIM and single marker regression, and demonstrate that the effect is consistently identified across the experiments. It is possible that the *T. macha* 4A resistance is conferred by multiple genes of small effect distributed over the approximately 12.2 cM region between markers BS00011060 and BS00164805. The additional recombinants within the 288 F_4_ lines and the high marker density in the present study, compared to the limited recombinants within the 43 DH lines studied by Steed et al. (2005), and in the 4A lines studied in the QTL combination field trials in the present study, may have fractionated *QFhs.jic*-*4AS* into multiple QTL within a small region. There is some evidence of this from the results obtained from the polytunnel trial when assessing disease severity, which identified three QTL peaks at BS00182960, TC93568 and Gwm192, and also from the JIC trial in 2012, which indicates two QTL conferring resistance to %FHB at Gwm165 and also at BS00022015-BS00068885. However, failure to resolve the QTL as a single gene may reflect the difficulty of accurately phenotyping FHB using only one score. This possibility is evidenced by the fact that the single-score data support a fractionated QTL while the integrated AUDPC score suggests a single QTL centring on marker TC93568. However, from the current data, it is not possible to determine whether there are multiple QTL or whether different loci have been detected as a consequence of unexplained variation in individual experiments. The generation of further recombinants and more detailed disease phenotyping of the lines using the greater accuracy that can be achieved in polytunnel trials are required to determine whether the phenotype observed from *QFhs.jic*-*4AS* is conferred by the additive effect of multiple QTL. Several previous studies have identified large effect QTLs that fractionate into multiple linked QTL when fine-mapping. This includes resistance against *Phytophthora infestans* in tomato (Johnson et al. 2012), the maize domestication QTL *teosinte branched 1* (Studer and Doebley 2011), and a malting quality QTL complex in barley (Gao et al. 2004). Other factors may have hindered resolution of *QFsh.jic*-*4AS* as a single gene. Accurate phenotyping of Type 1 resistance is recognised to be challenging because of confounding effects of Type 2 susceptibility in lines such as Hobbit sib. used in the present study. Furthermore, disease pressure was extremely high in all trials, as revealed by the high levels of DON, and this may have resulted in the fungus overcoming the resistance conferred by *QFsh.jic*-*4AS*. Additional, detailed phenotyping using reduced disease pressure may assist in resolving this issue.

The *QFhs.jic*-*4AS* was detected in approximately the same region, using both disease development (represented by AUDPC) and disease severity (represented by %FHB) measurements, in four independent phenotyping experiments. This suggests that the resistance can be considered to be stable, as it was expressed across different environments. Although previously identified as a Type 1 resistance, with no effect on disease spread following point inoculation, this data suggests that this resistance may have additional effects other than limiting initial colonisation (Type 1 resistance) as the resistance effect can be clearly observed when studying disease development over time (AUDPC). In addition, although the effects of the *QFhs.jic*-*4AS* QTL can be observed at earlier scoring times (data not shown), it appeared to have greatest effect when scoring visual symptoms after a relatively long period after inoculation (29–30 dpi) supporting the view that it also functions after initial infection.

There are some discrepancies between the trials that should be considered when choosing markers for selection of the *QFhs.jic*-*4AS* resistance. For example, BS00164805 would appear to be suitable as a proximal flanking marker based on the AUDPC data from all four trials and from the %FHB data from JIC and the polytunnel trial, providing no evidence of association with the resistance (*p* < 0.01) and hence suggesting that this marker is located immediately proximal to the resistance. However, BS00164805 provides a significant association with the %FHB scores generated from the CF field experiment, suggesting that this marker may be associated with the QTL based on this analysis. In addition, the % variance accounted for tends to increase for this marker compared to the proximal marker TC90601. It is possible that this marker has been positioned incorrectly by genetic mapping and that it should sit between TC90601 and Gwm192, which would be supported by synteny in the region (Fig. 2). However, it is also possible that this marker is providing further evidence for fractionation of *QFhs.jic*-*4AS*.

EST-derived SSRs (La Rota et al. 2005) and wheat KASP assay SNPs derived from transcript sequencing (Allen et al. 2011, 2013) were used to identify orthologues in syntenic regions within the fully sequenced genomes of *Brachypodium*, rice and sorghum. This enabled the identification of the region in these species that was orthologous to *QFhs.jic*-*4AS.* This region was then examined to identify further wheat iSelect SNPs based on their homology with these reference genomes. However, there is some evidence of a breakdown of synteny between the order of loci as determined by the wheat × *T. macha* genetic map of 4A developed in this study and the physical gene orders in the reference sequences (Fig. 2). A breakdown of co-linearity may be anticipated on chromosome 4A because it has undergone a significant number of rearrangements compared to the structure of related species. Previous studies have identified a peri-centromeric inversion involving a portion of the ancient long arm and the complete short arm, and interchanges with chromosomes 5A and 7B (Devos et al. 1995; Miftahudin et al. 2004). More recently, next-generation sequencing and synteny with *Brachypodium*, rice and sorghum was used to construct a chromosome 4A ‘genome-zipper’ with five syntenic segments (Hernandez et al. 2012). The QTL region in the present study lies partly within the syntenous chromosomal segment ‘A’ identified by Hernandez et al. (2012) from Bradi1g65190 to Bradi1g72092. The breakdown of gene order conservation within the QTL region and our inability to establish any co-linearity outside of the region, suggests that there may be a limitation in the use of synteny for further fine-mapping of *QFhs.jic*-*4AS*, particularly as the resistance may be controlled by multiple loci over the region.

In conclusion, we have demonstrated that Type 1 and Type 2 resistances can be combined in a highly susceptible background to provide an additive reduction in visual disease symptoms. However, caution should be exercised as this reduction in visual symptoms, may not be translated into a reduction in mycotoxin levels. To enhance the capabilities for marker assisted selection of the FHB Type 1 resistance *QFhs.jic*-*4AS,* we have developed additional recombinants and identified a number of SNP markers suitable for use by plant breeders. However, we were not able to locate and map the resistance as a single gene.

### Author contribution statement

CB undertook mapping, genotyping, phenotyping and writing of the manuscript, NG initiated production of QTL materials, AS undertook crossing and field work, ML undertook FHB trials in Tulln, NB and RR-G assisted with marker development, SH undertook mapping population development, and PN conceived and coordinated the work.

## Electronic supplementary material

Supplementary material 1 (BMP 1046 kb). Supplementary material 1 (BMP 1046 kb). Fig. S1: Histograms of predicted means for **a** AUDPC in JIC 2012, **b** %FHB in JIC 2012, **c** AUDPC in Polytunnel 2012, **d** %FHB in Polytunnel 2012, **e** AUPDC in JIC 2013, **f** %FHB in JIC 2013, AUDPC in CF 2013, and **g** %FHB in CF 2013

Supplementary material 2 (DOCX 17 kb)

Supplementary material 3 (DOCX 14 kb)

Supplementary material 4 (DOCX 16 kb)
