# Supplementary figures and images for: Mapping a Type 1 FHB resistance on chromosome 4AS of Triticum macha and deployment in combination with two Type 2 resistances
Source: Theor Appl Genet. 2015 Jun 4;128(9):1725–38. doi: 10.1007/s00122-015-2542-9 (PMC4540761; doi:10.1007/s00122-015-2542-9)

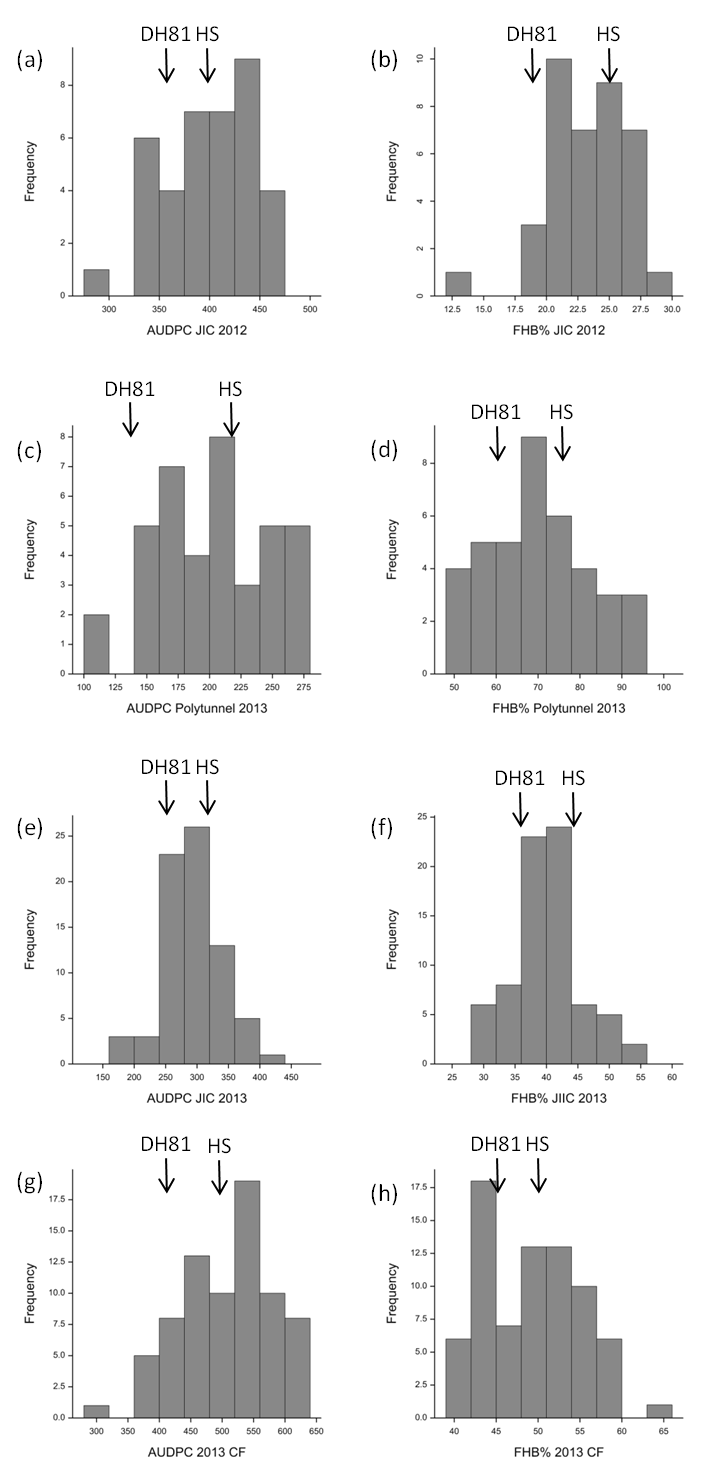

Supplement: Supplementary file 1 — Supplementary material 1 (BMP 1046 kb). Supplementary material 1 (BMP 1046 kb). Fig. S1: Histograms of predicted means for a AUDPC in JIC 2012, b %FHB in JIC 2012, c AUDPC in Polytunnel 2012, d %FHB in Polytunnel 2012, e AUPDC in JIC 2013, f %FHB in JIC 2013, AUDPC in CF 2013, and g %FHB in CF 2013 [file 122_2015_2542_MOESM1_ESM.bmp]
