# Supplementary material for: Mapping a Type 1 FHB resistance on chromosome 4AS of Triticum macha and deployment in combination with two Type 2 resistances
Source: Theor Appl Genet. 2015 Jun 4;128(9):1725–38. doi: 10.1007/s00122-015-2542-9 (PMC4540761; doi:10.1007/s00122-015-2542-9)
Supplement: Supplementary file 2 — Supplementary material 2 (DOCX 17 kb) [file 122_2015_2542_MOESM2_ESM.docx]

Table S1: Summary of primers for markers used for mapping Hobbit ‘sib.’ (HS) x DH81.

| Marker Name | Chromo. | Marker Set | Forward primers (and fluorophore) | Reverse primer |
| --- | --- | --- | --- | --- |
| BS00043286 | 4A | KASP | ACTTTTCAATGGAAATTTGATGATGTCT (FAM) / ACTTTTCAATGGAAATTTGATGATGTCC (VIC) | CACATCTTCAACGCGGATCACCAAA |
| BS00003914 | 4A | KASP | CCTCGAGGTCCCAGTTTCTTGAA (FAM) / CTCGAGGTCCCAGTTTCTTGAC (VIC) | CTTTACAAAAGCAGGATTTAGCGACTATAA |
| BS00001207 | 4A | KASP | GGGCGCCATGGAGACTCTGA (FAM) / GGCGCCATGGAGACTCTGG (VIC) | GTGGCTGCGCTGCTGCGAGAT |
| BS00003776 | 4A | KASP | GGGAATTGCAATTGGTGTTAAAGCC (FAM) / AGGGAATTGCAATTGGTGTTAAAGCT (VIC) | CAACAGTGACATCAAAATCTTGCTTCGTT |
| BS00022015 | 4A | KASP | GGATCCTGTTGTATGAGCTGCC (FAM) / GGATCCTGTTGTATGAGCTGCT (VIC) | TGCTCATCTGAATTGGAATGTTGGACAT |
| BS00068885 | 4A | KASP | ACGGCAGCAATTGTTTCACAGAAG (FAM) / CTACGGCAGCAATTGTTTCACAGAAA (VIC) | GGTTGAGCGATAGTCACTCCCATTT |
| BS00011060 | 4A | KASP | GCCAGCACTTTGGAGATAAGAAATCA (FAM) / CCAGCACTTTGGAGATAAGAAATCG (VIC) | GACTCAAACATCTGTCTTGACAAAGCAAA |
| BS00182960 | 4A | iSelect | CCTCAAAATCACTGAATGGTA (FAM) / CCTCAAAATCACTGAATGGTG (VIC) | ACGAGAGGATGAGTTCGACA |
| BS00011173 | 4A | KASP | GTACAGAATGGTCTAGCCA (FAM) / GCTGTACAGAATGGTCTAGCCG (VIC) | CCAATGGACTAGATGCGGGAGGAA |
| BS00113963 | 4A | KASP | ATTCATGTTGATCAGAAGTTAATT (FAM) / TTCATGTTGATCAGAAGTTAATC (VIC) | AGCGATTGTTGGGATTTGGA |
| TC93568 | 4A | EST-SSR | CAATCGTTGTCCAGAACCCT | CATGGGCATGTTAAGTGCAG |
| Gwm165 | 4A | SSR | TGCAGTGGTCAGATGTTTCC | CTTTTCTTTCAGATTGCGCC |
| Gwm192 | 4A | SSR | GGTTTTCTTTCAGATTGCGC | CGTTGTCTAATCTTGCCTTGC |
| TC90601 | 4A | EST-SSR | TGCTCGCTCTACTGCTGCT | CAGCACTCTCCTGCTACACG |
| BS00164805 | 4A | iSelect | GAAGGTGACCAAGTTCATGCT (FAM) / GAAGGTCGGAGTCAACGGATT (VIC) | GGATGTCAGGTTCATATCAACTCC |
| Wmc48 | 4A | SSR | GAGGGTTCTGAAATGTTTTGCC | ACGTGCTAGGGAGGTATCTTGC |
| BS00036472 | 4A | KASP | TATGACGACTGACTCACGGCC (FAM) / GTTATGACGACTGACTCACGGCT (VIC) | CTCTGGATGGGAACTGTAAGTCTGAT |
| BS00009974 | 4A | KASP | TGGTTTGCTCACTCAGCAG (FAM) / GCTTGGTTTGCTCACTCAGCAC (VIC) | ACGAATACATGGGGTTCCTCAGCTT |
| BS00011261 | 4A | KASP | ACTGTCTGTTACACAGCAGAGC (FAM) / CTACTGTCTGTTACACAGCAGAGT (VIC) | CTCCATCTTCAACGTTCCTGACCTT |
| BS00022816 | 4A | KASP | CATTGTGAGGTGCCAAGTGCTC (FAM) / CATTGTGAGGTGCCAAGTGCTG (VIC) | CTTGCGTGTGATCATGATGCCACTA |
| BS00003623 | 4A | KASP | AGCTACATATGTACCCGGTACTG (FAM) / AGCTACATATGTACCCGGTACTC (VIC) | GAACAGAGCATGCATGCATGCAGTT |
| BS00160015 | 7A | iSelect | ACATTACCTACGCCTTTCTCCG (FAM) / ACATTACCTACGCCTTTCTCCA (VIC) | TTCAGAGAGCTGTTGCCTGG |
| BS00022576 | 4B | KASP | AAGCATCGCACACATCAACGCC (FAM) / AAAGCATCGCACACATCAACGCT (VIC) | AGAGGTAGGCTGCGAGTGGCTT |
