# Supplementary material for: Mapping a Type 1 FHB resistance on chromosome 4AS of Triticum macha and deployment in combination with two Type 2 resistances
Source: Theor Appl Genet. 2015 Jun 4;128(9):1725–38. doi: 10.1007/s00122-015-2542-9 (PMC4540761; doi:10.1007/s00122-015-2542-9)
Supplement: Supplementary file 3 — Supplementary material 3 (DOCX 14 kb) [file 122_2015_2542_MOESM3_ESM.docx]

Table S2: Summary of markers screened against Hobbit ‘sib.’ and DH81 to identify polymorphic markers for mapping

| Marker system | Total no. of markers screened | No. of 4AS markers screened | No. of markers on 4AS polymorphic between Hobbit 'sib' and DH81^1^ | % polymorphic | No. of markers included in HS x DH81 map |
| --- | --- | --- | --- | --- | --- |
| Wheat SNP KASP Probes^1^ | 4253 | 115 | 20 | 17.4 | 14 |
| Wheat SNP iSelect^2^ | 73442 | 1443 | 83 | 5.8 | 2 |
| SSR^3^ | 39 | 39 | 9 | 23.1 | 3 |
| COS^4^ | 33 | 33 | 0 | 0.0 | 0 |
| EST-SSR^5^ | 26 | 26 | 2 | 7.7 | 2 |

^1^ LGC Genomics wheat genotyping panel (Allen et al. 2012)

^2^ Wheat 90,000 SNP iSelect HD Custom Genotyping Chip from Illumina.

^3^ 4AS SSRs (Goyal et al. 2005)

^4^ COS markers with orthology to genes in the 4.9 Mb Brachypodium distachyon region Bd1g68120 - Bd1g74310 (http://www.wgin.org.uk/resources/Markers/TAmarkers.php)

^5^ Wheat EST-SSRs with orthology to genes in the 11.4 Mb rice region Os03g07160 - Os03g26460 (La Rota et al. 2005)
