# Supplementary material for: Mapping a Type 1 FHB resistance on chromosome 4AS of Triticum macha and deployment in combination with two Type 2 resistances
Source: Theor Appl Genet. 2015 Jun 4;128(9):1725–38. doi: 10.1007/s00122-015-2542-9 (PMC4540761; doi:10.1007/s00122-015-2542-9)
Supplement: Supplementary file 4 — Supplementary material 4 (DOCX 16 kb) [file 122_2015_2542_MOESM4_ESM.docx]

Table S3: The effect of background variation in HS x DH81 recombinant lines on predicted mean AUDPC scores and %FHB scores, as

calculated by a single marker regression using general linear models.

| Marker | Chromosome | Trait |  | % Variance Accounted For | | | | | | |
| --- | --- | --- | --- | --- | --- | --- | --- | --- | --- | --- |
|  |  |  |  | 2012 JIC |  | 2013 JIC |  | 2013 Bawburgh |  | 2013 Polytunnel |
|  |  |  |  |  |  |  |  |  |  |  |
| BS00022576 | 4B | AUDPC |  | 0%, p=0.519 |  | 0%, p=0.519 |  | 1.4%, p=0.162 |  | 0%, p=0.351 |
| BS00022576 | 4B | % FHB 29-30 dpi |  | 0%, p=0.748 |  | 0%, p=0.748 |  | 0%, p=0.352 |  | 0%, p=0.812 |
| BS00160015 | 7A | AUDPC |  | 0%, p=0.430 |  | 0%, p=0.430 |  | 0%, p=0.352 |  | 7.4%, p=0.059 |
| BS00160015 | 7A | % FHB 29-30 dpi |  | 0.7%, p=0.227 |  | 0.7%, p=0.227 |  | 0%, p=0.935 |  | 1.5%, p=0.223 |
|  |  |  |  |  |  |  |  |  |  |  |
|  |  |  |  |  |  |  |  |  |  |  |
